# A 10-year interval of cardiovascular effects of albuterol in asthma management: Graphical review

**DOI:** 10.1016/j.crphar.2025.100236

**Published:** 2025-10-22

**Authors:** Ghulam H. Abbas, Faaizah Ahmed, Reda Iqbal, Fathimathul Henna, Edmon R. Khouri, Frank W.J.M. Smeenk, Sjaak Pouwels

**Affiliations:** a Faculty of Medicine, Ala-Too International University, Bishkek, Kyrgyz Republic; b School of Medicine, Dubai Medical College, Dubai, United Arab Emirates; c School of Medicine, University of Jordan, Amman, Jordan; d Department of Medical Education, Catharina Hospital Eindhoven, the Netherlands; e School of Health Professions Education, Maastricht University, the Netherlands; f Department of Surgery, University Hospital OWL of Bielefeld University - Campus Klinikum Lippe, Detmold, NRW, Germany; g Department of Intensive Care Medicine, Elisabeth-Tweesteden Hospital Tilburg, the Netherlands

**Keywords:** Cardiovascular medicine, Albuterol, Asthma management, Airflow obstruction, Pulmonary disease, Cardiology

## Abstract

Asthma is a widespread chronic respiratory disease that requires effective management to reduce exacerbations and improve patient outcomes. Albuterol (salbutamol), a short-acting beta-2-agonist (SABA), remains a mainstay treatment for acute symptom relief due to its rapid bronchodilatory effect. However, accumulating evidence over the past decade has raised concerns about its cardiovascular safety, particularly in vulnerable populations such as children, elderly individuals, and those with underlying cardiac conditions. This review synthesizes clinical findings from the last ten years (2015–2025) evaluating the cardiovascular effects of albuterol, including tachycardia, arrhythmias, QTc prolongation, and hypotension. Literature across PubMed, Cochrane, and Google Scholar was analyzed to assess frequency, severity, and risk factors associated with these events. Notably, intravenous administration was associated with markedly higher rates of adverse effects, while inhaled formulations remained safer with moderate risk. Pediatric patients on continuous therapy showed increased susceptibility to electrolyte imbalances and hypotension. Although alternatives like levalbuterol demonstrated a reduced cardiovascular risk profile, they were linked with increased healthcare costs and longer hospital stays. The review highlights the importance of risk stratification, personalized dosing, and enhanced monitoring, particularly in high-risk groups, to maximize the therapeutic benefits of SABAs while minimizing cardiovascular harm. Overall, the findings underscore the need for ongoing pharmacovigilance and tailored clinical decision-making when prescribing albuterol in asthma care.

## Highlights

• The review evaluates cardiovascular side effects of albuterol over the past 10 years.
• In adults tachycardia and QTc Interval Prolongation were frequently found.
• In pediatric ICU patients changes in diastolic blood pressure are of significant concern.
• Levalbuterol can be a good alternative with less cardiovascular side effects.

## 1 Introduction and clinical context

Asthma remains one of the most prevalent chronic respiratory diseases globally, affecting approximately 339 million people across all age groups, ethnicities, and socio-economic backgrounds (Khuman et al., 2024). Its incidence continues to rise steadily due to environmental factors, urbanization, and changing lifestyle patterns, thereby imposing substantial healthcare burdens worldwide (Scott et al., 2022). Albuterol, also known as salbutamol, is classified as a short-acting beta-agonist (SABA) and is widely recognized as a cornerstone treatment for rapid symptomatic relief during acute asthma exacerbations (Nielsen et al., 2022). Its immediate bronchodilatory effects, resulting from potent relaxation of bronchial smooth muscles, have solidified its status as a first-line therapy for the emergency management of acute bronchospasm and as a rescue medication in chronic asthma management protocols (Shah Amran et al., 2023).

However, over the past decade, mounting clinical and epidemiological evidence has highlighted significant concerns about the cardiovascular safety of albuterol (Reddy et al.; Mendes et al., 2015; Wisecup et al., 2015; PubChem). Cardiovascular adverse events, previously perceived as relatively uncommon or mild, have now been documented with increasing frequency and severity, prompting a critical reassessment of clinical practices involving albuterol use (Elsevier – Drug Monograph). These cardiovascular concerns are particularly pronounced in vulnerable populations such as elderly patients, who often have diminished cardiac reserve, pediatric patients with immature cardiovascular systems, and individuals with pre-existing cardiovascular diseases including ischemic heart disease, heart failure, hypertension, and congenital cardiac anomalies (DrugBank Online, 2024; Medscape, 2020; Payares Salamanca et al., 2020).

Reported cardiovascular side effects encompass a broad spectrum of clinical presentations, including tachycardia, palpitation, QT interval prolongation, arrhythmias (notably supraventricular tachycardia and ventricular arrhythmias such as torsades de pointes), hypotension, myocardial ischemia, and various electrolyte imbalances, especially hypokalemia (Elsevier – Drug Monograph; DrugBank Online, 2024; Medscape, 2020; Payares Salamanca et al., 2020; LaForce et al., 2016; Rohr et al., 1986; Phumeetham et al., 2015; Saadia et al., 2015). Tachycardia is among the most frequently observed side effects and can exacerbate underlying cardiac disorders, leading to increased morbidity and, in severe cases, mortality (Lindquist and Cooper, 2013). QT interval prolongation, another major concern, significantly heightens the risk of potentially lethal arrhythmias, necessitating careful patient selection and monitoring during therapy (Ma et al., 2022). Additionally, chronic or excessive use of albuterol can lead to significant electrolyte disturbances, with hypokalemia further compounding cardiovascular risk and arrhythmogenic potential (Edgell et al., 2016).

**Fig. 1:**
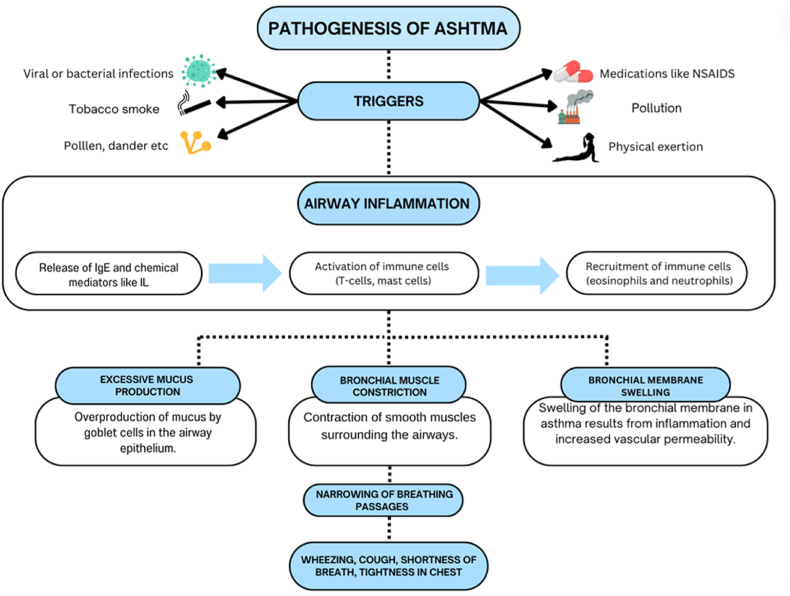
Pathogenesis of Asthma (Reddy et al.; Mendes et al., 2015; Wisecup et al., 2015; PubChem).

Given these concerns, clinicians must adopt a more cautious and individualized approach to prescribing albuterol, taking into account the patient's comprehensive cardiovascular risk profile, medical history, concurrent medications, and individual susceptibility to adverse effects (The effect of nebulized salbutamol on serum potassium and blood sugar level of asthmatic patients - grantha library; Johnson et al., 2024). Consequently, current guidelines and clinical practices are increasingly advocating for prudent prescribing, meticulous patient monitoring, and consideration of safer alternatives such as levalbuterol, particularly in high-risk patient cohorts (Kearns et al., 2022; Shebli et al., 2024; Peng et al., 2023; Marques and Vale, 2022; Moore et al., 2019; Najout et al., 2020; Brunetti et al., 2015) (Fig. 1).

## 2 Mechanisms of albuterol on cardiovascular effects

Albuterol exerts its therapeutic action primarily through the selective activation of β2-adrenergic receptors, which are widely distributed in bronchial smooth muscle cells, cardiac tissues, vascular smooth muscle, and skeletal muscle (Reddy et al.). Activation of these receptors triggers an intracellular cascade mediated by cyclic adenosine monophosphate (cAMP), leading to relaxation of bronchial smooth muscle and subsequent relief of bronchoconstriction, thereby improving airflow and reducing acute respiratory distress (Reddy et al.; Mendes et al., 2015; Wisecup et al., 2015; PubChem; Elsevier – Drug Monograph; DrugBank Online, 2024; Medscape, 2020; Payares Salamanca et al., 2020). However, this therapeutic mechanism is intricately linked with several unintended cardiovascular effects resulting from β2 receptor stimulation in cardiovascular tissues (Edgell et al., 2016; The effect of nebulized salbutamol on serum potassium and blood sugar level of asthmatic patients - grantha library; Johnson et al., 2024; Kearns et al., 2022).

Upon stimulation by albuterol, β2-adrenergic receptors within cardiac tissue significantly elevate intracellular cAMP, thereby activating protein kinase A (PKA) (Iramain et al., 2019). This enzyme-mediated cascade enhances calcium influx and release within cardiomyocytes, consequently producing notable chronotropic (increased heart rate) and inotropic (increased cardiac contractility) effects (Katsunuma et al., 2019). Such effects can be beneficial in certain clinical contexts, yet in patients with compromised cardiovascular function, these effects may lead to exacerbation of ischemic conditions, arrhythmogenicity, and compromised cardiac stability (Katsunuma et al., 2019).

Peripheral vasodilation, another consequence of β2 receptor activation within vascular smooth muscle, can result in reflex tachycardia due to compensatory sympathetic nervous system activation aimed at maintaining adequate cardiac output and systemic blood pressure (Burggraaf et al., 2001). This reflexive mechanism further amplifies cardiac stress, particularly in patients with pre-existing cardiac conditions (Phillips et al., 1980a).

Additionally, albuterol administration frequently induces hypokalemia through β2 receptor-mediated stimulation of the sodium-potassium ATPase pumps in skeletal muscles, driving potassium intracellularly and significantly lowering serum potassium levels (Phillips et al., 1980a). This electrolyte imbalance profoundly heightens the risk of cardiac arrhythmias, including QT prolongation and torsades de pointes, by altering myocardial excitability and repolarization dynamics (Saadia et al., 2015; Lindquist and Cooper, 2013; Ma et al., 2022; Edgell et al., 2016; The effect of nebulized salbutamol on serum potassium and blood sugar level of asthmatic patients - grantha library). The resultant cardiac electrical instability is particularly dangerous in vulnerable patient populations or those concurrently using medications that affect myocardial conduction or prolong QT intervals (Gomes et al., 2020).

Individual genetic variations significantly influence susceptibility to these cardiovascular side effects. Polymorphisms in genes encoding β2-adrenergic receptors and metabolic enzymes such as sulfotransferases and cytochrome P450 isoforms critically affect receptor sensitivity, drug metabolism, and pharmacokinetic profiles (Lin et al., 2019). Patients with specific genetic variants may exhibit heightened receptor sensitivity, prolonged receptor activation, or altered metabolic clearance, consequently experiencing more pronounced cardiovascular effects at standard therapeutic doses (Albuterol.). This genetic variability underscores the importance of personalized medicine, advocating for tailored therapeutic approaches based on genetic profiling, particularly in high-risk patients (Albuterol: uses, interactions, mechanism of action DrugBank online).

**Fig. 2:**
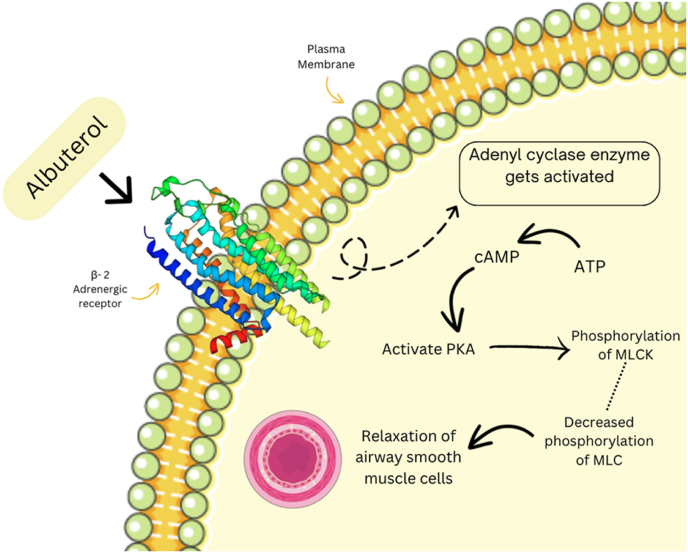
Mechanism of action of Albuterol (Payares Salamanca et al., 2020; LaForce et al., 2016; Rohr et al., 1986; Phumeetham et al., 2015; Saadia et al., 2015; Lindquist and Cooper, 2013; Ma et al., 2022; Edgell et al., 2016).

Overall, understanding these complex and interconnected mechanisms underlying albuterol's cardiovascular effects is crucial in devising therapeutic strategies aimed at maximizing respiratory benefits while minimizing cardiovascular risks (Proventil and Ventolin). Clinicians should therefore integrate comprehensive mechanistic insights with patient-specific factors to optimize asthma management protocols and ensure maximal patient safety (Fig. 2).

## 3 Evidence from literature (2015–2025)

Over the past decade, extensive research—including randomized controlled trials (RCTs), observational cohort studies, case reports, meta-analyses, and comprehensive systematic reviews—has rigorously evaluated the cardiovascular risks associated with albuterol usage (PubChem; Elsevier – Drug Monograph; DrugBank Online, 2024; Medscape, 2020; Payares Salamanca et al., 2020; LaForce et al., 2016; Rohr et al., 1986; Phumeetham et al., 2015; Saadia et al., 2015; Lindquist and Cooper, 2013; Ma et al., 2022; Edgell et al., 2016; The effect of nebulized salbutamol on serum potassium and blood sugar level of asthmatic patients - grantha library; Johnson et al., 2024). Tachycardia has consistently emerged as one of the most frequently reported adverse effects, documented in approximately 16 % of patients receiving standard-dose inhaled albuterol, with this figure significantly escalating to nearly 51 % in individuals receiving intravenous administration or higher-than-standard dosing (The effect of nebulized salbutamol on serum potassium and blood sugar level of asthmatic patients - grantha library; Johnson et al., 2024; Kearns et al., 2022; Shebli et al., 2024; Peng et al., 2023; Marques and Vale, 2022; Moore et al., 2019; Najout et al., 2020). Furthermore, tachycardia incidence rates appear to be exacerbated in certain vulnerable populations, such as pediatric patients, elderly individuals, and patients with underlying cardiovascular conditions (Shebli et al., 2024; Peng et al., 2023; Marques and Vale, 2022; Moore et al., 2019; Najout et al., 2020; Brunetti et al., 2015; Iramain et al., 2019; Katsunuma et al., 2019; Burggraaf et al., 2001; Phillips et al., 1980a; Gomes et al., 2020; Lin et al., 2019; Albuterol.).

The phenomenon of QT interval prolongation has also been extensively documented across numerous clinical studies and individual case reports. QT prolongation is particularly concerning due to its association with severe arrhythmogenic events, including torsade de pointes, a life-threatening polymorphic ventricular tachycardia (Morgan et al., 1986). Pediatric populations are notably susceptible, as indicated by several pediatric-focused studies demonstrating increased hospitalization rates and therapy cessation directly linked to albuterol-induced QT prolongation and arrhythmias (Ventolin side effects: common, severe, long term).

Comparative analyses between the commonly prescribed racemic form of albuterol and its stereoisomer, levalbuterol, have provided significant insights into differential cardiovascular risk profiles (Ventolin side effects: common, severe, long term). Multiple studies indicate that levalbuterol, due to its selective isomeric structure, exhibits a substantially reduced incidence of cardiovascular side effects (Albuterol side effects: common, severe, long term). Notably, observational data and randomized comparative trials have demonstrated fewer episodes of tachycardia and lower rates of significant QT interval prolongation with levalbuterol compared to racemic albuterol (Ventolin side effects: common, severe, long term). Despite these promising safety data, levalbuterol's higher acquisition costs, limited availability in certain regions, and increased hospital length of stay observed in some clinical settings have tempered widespread adoption, emphasizing a need for individualized therapeutic decisions (Albuterol side effects: common, severe, long term; Albuterol: 7 things you should know).

Pharmacovigilance databases and real-world observational studies further substantiate the clinical concerns raised by randomized clinical trials (Albuterol side effects: common, severe, long term). The Food and Drug Administration's Adverse Event Reporting System (FAERS) and the World Health Organization's pharmacovigilance database, VigiBase, both consistently report heightened cardiovascular risk signals associated with higher-dose or chronic albuterol therapy (Lin et al., 2019; Albuterol.; Albuterol: uses, interactions, mechanism of action DrugBank online; Proventil and Ventolin; Morgan et al., 1986; Ventolin side effects: common, severe, long term). These pharmacovigilance analyses reveal an increased incidence of reported tachycardia, cardiac arrhythmias, hypertension, and electrolyte disturbances, notably hypokalemia, with increasing cumulative exposure and prolonged duration of therapy (Peng et al., 2023; Marques and Vale, 2022).

Collectively, the evidence from the past decade underscores the critical necessity for clinicians to balance the undeniable therapeutic efficacy of albuterol against its associated cardiovascular risks, particularly when considering therapy in higher-risk patient populations (Moore et al., 2019; Najout et al., 2020; Brunetti et al., 2015; Iramain et al., 2019; Katsunuma et al., 2019; Burggraaf et al., 2001; Phillips et al., 1980a; Gomes et al., 2020; Lin et al., 2019; Albuterol.). The depth of available evidence provides a robust foundation for reevaluating current prescribing guidelines and practices, ensuring maximal therapeutic benefit while minimizing potential cardiovascular harm (Table 2).

**Table 1:** Summary of cardiovascular side effects associated with albuterol.

Side Effect	Incidence Rate (%)	Administration Route	Statistical Significance
Tachycardia	16 (inhaled), 51 (IV)	Inhaled, Intravenous	p < 0.001 (IV vs Inhaled)
QTc Interval Prolongation	Not specified	Inhaled	Significant in adults (withdrawals noted)
Hypotension	90 (during continuous therapy)	Continuous nebulization	p < 0.05 (compared to baseline)
Hypokalemia	15.2 (in PICU patients)	Continuous nebulization	p < 0.001 (compared to placebo)
Tremors	9	Inhaled	Not specified

**Table 2:** Comparative analysis of albuterol and related bronchodilator therapies: Efficacy, safety, and adverse effects.

Study	Objective	n	Outcome	Follow-up	Adverse Effects	Statistical Analysis
Yogesh N.V. Reddy et al.	To assess whether inhaled β-adrenergic agonist albuterol improves pulmonary vascular function during exercise in patients with HFpEF without increasing left heart filling pressures.	30	Albuterol improved exercise pulmonary vascular resistance without increasing pressures.	Not specified (acute trial only)	No significant adverse effects, albuterol decreased RA pressure and systemic blood pressure.	Used ANOVA, paired t-tests for specific pair differences, and Tukey's post-hoc tests for multiple comparisons, with significance set at significance at P < 0.05 for bronchodilation and P < 0.005 for vasodilation
Mendes et al.	To determine if a single dose of an inhaled glucocorticosteroid (ICS), specifically mometasone, acutely potentiates albuterol-induced airway smooth muscle relaxation in patients with moderate asthma with airflow obstruction.	15	Mometasone significantly potentiated bronchodilator response to albuterol.	Suggests further studies	None reported; typical exclusion criteria included cardiovascular disease and respiratory infections.	The primary endpoint was the change in pulmonary vascular resistance during exercise. Statistical comparisons between the albuterol and placebo groups were made using appropriate statistical tests (not specified in the abstract). Significance levels were reported for the primary endpoint (P = 0.003) and other outcomes (all P < 0.01), indicating statistically significant differences favoring albuterol over placebo.
Wisecup et al.	To assess the prevalence and risk factors for diastolic hypotension in pediatric patients receiving continuous albuterol nebulization for status asthmaticus.	152	90 % of patients developed diastolic hypotension correlated with albuterol dosing.	Not specified	Diastolic hypotension most common, related to albuterol dosing.	reduced risk of hypotension were 82 % lower with fluid boluses (OR: 0.18, p = 0.005). Higher albuterol doses increased hypotension risk (OR: 1.12 per 10 mg increase).
Craig LaForce et al.	Compare efficacy and safety of albuterol dry powder inhaler (MDPI) vs. placebo in children with asthma.	184	Albuterol MDPI significantly improved PPFEV1 over placebo.	3 weeks	Well tolerated with effects lasting several hours.	Mean difference of 25 % in PPFEV1 AUC0-6 (p < 0.0001) in favor of albuterol; effect onset within 5 min
Phumeetham et al.	To examine the efficacy and safety of high-dose continuous albuterol nebulization in children admitted to the PICU with status asthmaticus.	42	High-dose continuous albuterol associated with low mechanical ventilation rates.	During therapy	Minor hemodynamic changes like increased HR, decreased DBP, and mild hypokalemia.	Descriptive data were reported as means, and the significance of differences between variables was assessed using a two-tailed Student's t-test or the Mann-Whitney *U* test for non-normally distributed variables. A p-value of <0.05 was considered statistically significant
Edgell et al.	To examine the short-term cardiovascular and autonomic effects of inhaled salbutamol in healthy adults.	12	Increased heart rate and MSNA, no effect on arterial stiffness or blood pressure.	Short-term effects after dosing	None directly reported; increased MSNA suggests potential long-term cardiovascular risks.	Two-way repeated measures ANOVA with post hoc Student-Neuman-Keuls tests, significance set at p ≤ 0.05
Brunetti et al.	Compare clinical outcomes and treatment costs between levalbuterol and albuterol in COPD/asthma patients.	112	Levalbuterol and albuterol had similar outcomes, but levalbuterol incurred higher costs.	Hospital stay	No significant difference in adverse effects between groups.	Statistical methods included Student's t-test, Wilcoxon rank sum test, chi-square, Fisher's exact test, and generalized linear models with log link functions.
Kearns et al.	To compare the bronchodilator, systemic beta2-agonist, cardiovascular, and adverse effects of salbutamol 200 μg and budesonide/formoterol 200/6 μg in stable asthma patients.	39	Salbutamol had greater initial bronchodilation compared to budesonide/formoterol.	420 min	Two patients withdrew due to adverse events; salbutamol caused more adverse events.	Model-based estimated difference in FEV1 with 95 % CI: 0.12 (−0.25 to 0.02) L, p = 0.088. Other analysis of FEV1 over time was done using repeated measures analysis.
Moore et al.	To assess the acute effects of salbutamol on systemic vascular function in asthmatic and control subjects.	14 asthmatics, 14 controls (Phase I); 10 asthmatics, 10 controls (Phase II)	Reduced FMD and increased arterial stiffness in asthmatics after salbutamol; no significant effects in controls.	Phase I: 15 min after inhalation; Phase II: 2 min into hand submersion in body-temperature and ice water	Increased heart rate in both asthmatics and controls, increased arterial stiffness in asthmatics.	Three-way repeated measure ANOVA
Farooq et al.	To evaluate the effect of nebulized salbutamol on serum potassium and blood sugar levels in asthmatic patients who have normal levels at baseline.	100	Salbutamol significantly decreased serum potassium and increased blood sugar levels after administration.	Potassium and blood sugar levels were measured at baseline, 30 min, and 60 min after salbutamol administration.	Significant hypokalemia and hyperglycemia were observed 1 h after nebulization.	One-way ANOVA was used to compare differences in means across the three time points, with significant findings (p < 0.05) reported for changes in both potassium and glucose levels after 1 h.
Ghabally et al.	To summarize the effects of racemic albuterol versus levalbuterol on heart rate in asthmatic children, comparing their cardiac side effects and overall efficacy.	1002	Primary outcome: Heart rate changes, Secondary outcomes: Respiratory rate, FEV1 peak percent changes, potassium serum levels, SpO2 peak changes, asthma score, and adverse effects.	Different studies measured outcomes at varied time points.	Nausea, vomiting, headache, jitteriness, drop in potassium levels, lightheadedness, tremors, tachycardia, high temperature. No significant differences in adverse effects between levalbuterol and albuterol.	Meta-analysis using mean difference (MD) and risk ratio (RR) with a 95 % confidence interval (CI). Heterogeneity was assessed using forest plots, Chi^2^, and I^2^. A random-effects model was applied where heterogeneity was significant. Sensitivity analyses were conducted to test the robustness of the findings.
Ma et al.	To systematically assess the potential adverse events (AEs) associated with salbutamol through a meta-analysis of randomized controlled trials (RCTs).	58 RCTs involving 12,961 participants.	Pooled incidence of total AEs: 34 %, severe AEs: 2 %, treatment discontinuation: 3 %, most common AEs: palpitations/tachycardia (16 %), tremors, and anxiety.	Varies by study; detailed follow-up times are not specified for each included trial but range from 1 day to 52 weeks.	Most frequently observed AEs: palpitations or tachycardia, tremors, anxiety. Intravenous salbutamol users and premature labor cases had higher AE rates.	Random-effects model was used to calculate pooled incidence. Subgroup analysis examined variations based on indications (asthma, COPD, premature labor) and formulation (inhaled, oral, intravenous). Sensitivity analysis and tests for publication bias (Egger's test and Begg's test) were also performed.

## 4 Clinical implications

In light of the robust evidence indicating significant cardiovascular risks associated with albuterol usage, clinicians must adopt comprehensive and proactive cardiovascular risk management protocols prior to initiating therapy, especially in chronic or high-dose scenarios (Albuterol side effects: common, severe, long term). It is imperative that initial patient evaluations include detailed cardiovascular risk profiling, assessing factors such as patient age, history of cardiovascular disease, family history of sudden cardiac death or arrhythmias, and concomitant use of other QT-prolonging medications (Albuterol side effects: common, severe, long term).

Electrolyte disturbances, particularly hypokalemia, are common and well-documented sequelae of high-dose or prolonged albuterol therapy and are intrinsically linked to heightened arrhythmia risk (Fagbuyi et al., 2016). Consequently, routine assessment and correction of serum potassium levels should be standard practice, particularly in high-risk groups (Persch et al., 2024). Additionally, regular electrocardiographic (ECG) monitoring is strongly recommended in patients undergoing prolonged albuterol therapy, patients with existing cardiac comorbidities, and those receiving concurrent medications that may further predispose them to arrhythmias (Fagbuyi et al., 2016).

Current asthma management guidelines should explicitly incorporate these cardiovascular risk considerations into their therapeutic algorithms, advocating a minimal effective dose strategy to mitigate unnecessary cardiovascular exposure (Warraich et al., 2023). Clinicians are encouraged to critically evaluate the necessity of continued high-dose therapy, exploring the possibility of dose tapering where clinically appropriate (Persch et al., 2024).

Given the documented safer cardiovascular profile of levalbuterol, consideration of this alternative medication should be strongly advocated, particularly in high-risk patient groups identified through risk stratification (Warraich et al., 2023). While economic constraints and availability issues currently limit the widespread adoption of levalbuterol, healthcare policymakers and clinicians must collaborate to evaluate cost-effectiveness comprehensively, balancing economic considerations with patient safety and clinical outcomes (Albuterol side effects: common, severe, long term).

Structured decision-making frameworks, integrating pharmacological insights, real-world safety data, and individualized patient assessments, will serve as crucial clinical tools (Albuterol side effects: common, severe, long term). Such frameworks facilitate tailored prescribing practices, ensuring optimal therapeutic efficacy while prioritizing patient cardiovascular safety (Warraich et al., 2023). Continuous education of healthcare professionals regarding these risks and management strategies is essential, emphasizing the importance of integrating pharmacovigilance data into clinical decision-making processes (De Vries et al., 2008). Ultimately, the goal is to optimize therapeutic efficacy while simultaneously minimizing cardiovascular risks, thus enhancing patient safety in the broader context of asthma management (Table 1).

## 5 Future directions

Future investigations must prioritize large-scale, randomized controlled clinical trials explicitly designed to evaluate the cardiovascular safety profile associated with short-acting beta-agonists (SABAs), particularly albuterol (Light, 1979). Current literature predominantly comprises observational studies, pharmacovigilance analyses, and smaller randomized trials, limiting comprehensive risk assessment (Le Bourgeois et al., 1990). Robust, adequately powered randomized trials, inclusive of diverse patient demographics such as elderly, pediatric, and patients with multiple cardiovascular comorbidities, will significantly enhance our understanding of cardiovascular risks (Le Bourgeois et al., 1990). Such studies should aim to systematically measure and document cardiovascular endpoints, including detailed monitoring of heart rate variability, QT interval prolongation, arrhythmia incidence, myocardial ischemia events, and electrolyte disturbances. Incorporating real-world clinical scenarios within these studies will further strengthen their external validity and practical applicability (Misra et al., 1981).

Pharmacogenomic research represents another critical frontier, promising substantial advancements in personalized medicine (Le Bourgeois et al., 1990). Identifying genetic biomarkers or polymorphisms associated with increased susceptibility to cardiovascular adverse effects will facilitate the development of tailored treatment strategies (Misra et al., 1981). Genetic variations influencing β2-adrenergic receptor sensitivity, metabolic pathways including hepatic enzymes such as CYP450 isoforms, and sulfotransferase activity represent critical targets for research (Phillips et al., 1980b). By leveraging pharmacogenomic insights, clinicians can proactively identify at-risk individuals, enabling optimized therapeutic regimens through adjusted dosing strategies, alternative drug selection, or enhanced cardiovascular monitoring protocols (Rebuck and Contreras, 1982).

Enhancing pharmacovigilance mechanisms through the integration of advanced digital healthcare systems and real-time adverse event reporting platforms will significantly bolster the capability to rapidly detect, analyze, and mitigate emerging cardiovascular safety signals (Salengros et al., 2004). Artificial intelligence (AI) and machine-learning technologies can be employed to analyze large-scale pharmacovigilance datasets, recognizing early patterns and subtle signals of cardiovascular harm associated with SABA use (Salengros et al., 2004). Such technological advancements will facilitate prompt clinical responses and adjustments in therapeutic recommendations, ultimately enhancing patient safety.

Additionally, regulatory bodies must adopt dynamic frameworks for continuously updating treatment guidelines, prescribing information, and safety warnings (Phillips et al., 1980b; Rebuck and Contreras, 1982). Regularly incorporating emerging evidence from ongoing pharmacovigilance, clinical trials, and real-world observational studies into guideline development will ensure healthcare providers have current and precise recommendations for clinical practice (Misra et al., 1981; Phillips et al., 1980b; Rebuck and Contreras, 1982; Salengros et al., 2004; Scalabrin et al., 1996; Cherney, 2022). Education initiatives targeting healthcare professionals should concurrently emphasize awareness of cardiovascular risks, guideline adherence, and optimal monitoring strategies during SABA therapy (Light, 1979; Le Bourgeois et al., 1990; Misra et al., 1981; Phillips et al., 1980b; Rebuck and Contreras, 1982).

Finally, economic evaluations assessing the cost-effectiveness and healthcare resource implications of alternative therapies, such as levalbuterol, compared to racemic albuterol, should be undertaken (Scalabrin et al., 1996). These evaluations will inform healthcare policy, enabling balanced decisions that weigh cardiovascular safety, clinical outcomes, and economic feasibility (Cherney, 2022). Overall, such comprehensive, multidimensional research efforts and policy updates will be pivotal in advancing asthma care, prioritizing patient safety, and maximizing therapeutic efficacy in clinical practice (Table 3).

**Table 3:** Risk assessment and monitoring recommendations for albuterol use.

Risk Factor	Assessment Method	Monitoring Frequency
Pre-existing cardiovascular disorders	Medical history review	At each visit
Blood Pressure	Routine blood pressure checks	At each visit
Heart Rate	Continuous monitoring (wearable tech)	Daily during therapy
Electrolyte Levels	Serum potassium tests	Weekly during continuous therapy
Patient Compliance	Patient feedback and questionnaires	At each visit

## Credit author statement

Initial idea: **GA,**

Literature Search: **GA, FA, RI, FH, EK, FS, SP**.

Data Analysis: **GA, FA, RI, FH, EK, FS, SP**.

Writing the manuscript: **GA, FA, RI, FH, EK, FS, SP**.

Final Approval: **GA, FA, RI, FH, EK, FS, SP**.

## Funding

None.

## Declaration of competing interest

The authors declare that they have no known competing financial interests or personal relationships that could have appeared to influence the work reported in this paper.

## Data Availability

Data will be made available on request.
